# Are area-based initiatives able to improve area safety in deprived areas? A quasi-experimental evaluation of the Dutch District Approach

**DOI:** 10.1186/s12889-015-2027-4

**Published:** 2015-07-28

**Authors:** Daniëlle Kramer, Birthe Jongeneel-Grimen, Karien Stronks, Mariël Droomers, Anton E. Kunst

**Affiliations:** Department of Public Health, Academic Medical Centre, University of Amsterdam, Amsterdam, The Netherlands

**Keywords:** Safety, Victimization, Disorder, Area-based initiative, Neighbourhood regeneration, Quasi-experimental evaluation

## Abstract

**Background:**

Numerous area-based initiatives have been implemented in deprived areas across Western-Europe with the aim to improve the socio-economic and environmental conditions in these areas. Only few of these initiatives have been scientifically evaluated for their impact on key social determinants of health, like perceived area safety. Therefore, this study aimed to assess the impact of a Dutch area-based initiative called the District Approach on trends in perceived area safety and underlying problems in deprived target districts.

**Methods:**

A quasi-experimental design was used. Repeated cross-sectional data on perceived area safety and underlying problems were obtained from the National Safety Monitor (2005–2008) and its successor the Integrated Safety Monitor (2008–2011). Study population consisted of 133,522 Dutch adults, including 3,595 adults from target districts. Multilevel logistic regression analyses were performed to assess trends in self-reported general safety, physical order, social order, and non-victimization before and after the start of the District Approach mid-2008. Trends in target districts were compared with trends in various control groups.

**Results:**

Residents of target districts felt less safe, perceived less physical and social order, and were victimized more often than adults elsewhere in the Netherlands. For non-victimization, target districts showed a somewhat more positive change in trend after the start of the District Approach than the rest of the Netherlands or other deprived districts. Differences were only statistically significant in women, older adults, and lower educated adults. For general safety, physical order, and social order, there were no differences in trend change between target districts and control groups.

**Conclusions:**

Results suggest that the District Approach has been unable to improve perceptions of area safety and disorder in deprived areas, but that it did result in declining victimization rates.

## Background

A recent review revealed that in the past decade, numerous area-based initiatives (ABIs) have been implemented in deprived areas across Western-Europe [1]. ABIs are defined as large-scale programmes that aim to improve both the physical and social environmental conditions of deprived areas, as well as the socio-economic position of its residents. These initiatives have the potential to improve health and reduce health inequalities by improving key social determinants of health, such as employment, housing, and area safety [2–4]. However, only few evaluation studies have been able to assess the impact of ABIs on health [2, 3]. Where impacts have been assessed, health improvements were often small [3]. It has been suggested that this lack of evidence is due to the long time needed to detect health impacts [2]. An alternative strategy may therefore be to assess the impact of ABIs on key social determinants of health, such as perceived area safety, which may change more quickly in response to local policies.

Residents of deprived areas feel less safe than residents of non-deprived areas [5, 6]. Perceived lack of area safety has been identified as a risk factor for health [7, 8]. Safety concerns may induce psychological stress or may keep people from going outdoors, which limits social interaction and physical activity [7, 8]. Thus, from a public health perspective it is important to improve perceptions of area safety in deprived areas. However, perceived area safety is a complex factor and its potential causes have extensively been studied and discussed. Traditionally, researchers have focused on criminal victimization as the main cause of safety concerns, but safety concerns are far more widespread than crime, suggesting additional causes [9–11]. Many researchers have argued that safety concerns are not specifically related to the incidence of crime, but rather reflects a general anxiety or broader perceptions of the social and physical environment [7]. The incivilities thesis posits that safety concerns are the result of disorder, i.e., incivilities [12]. Signs of disorder may be physical (e.g., litter, graffiti) or social (e.g., public drinking, drug use, nuisance from youth). Residents may interpret disorder as a sign that fellow residents and officials are unable or unwilling to solve problems. As a result, residents may feel personally at risk of more serious crime, causing them to feel unsafe. There is strong quantitative and qualitative evidence for the association between disorder and safety concerns [7, 8].

In addition, safety concerns are suggested to be the result of poor neighbourhood conditions, although the evidence here is less consistent [7, 8, 13]. The most influential theory in this field has been Cozens’ Crime Prevention Through Environmental Design (CPTED) [14]. One of the concepts central to CPTED is surveillance [14]. Poorly designed areas (e.g., areas that are poorly lit, isolated, or where sight-lines are obstructed by vegetation or buildings) provide limited options for surveillance from fellow residents. This may cause people to feel more vulnerable to crime, resulting in safety concerns. Strong social networks may safeguard against the fear resulting from poor physical conditions by reducing feelings of vulnerability. Limited surveillance options may also increase crime and disorder by increasing the amount of potential hiding places for offenders and reducing the chances of being caught. Again, strong social networks may mitigate this effect by signalling to offenders that residents are willing to intervene for the community’s benefit [15].

ABIs have the potential to improve perceptions of area safety in deprived areas, either by tackling underlying problems of crime and disorder, or by improving physical and social neighbourhood conditions. A recent review has identified only two ABIs that have been evaluated for their impact on safety concerns and related safety problems: the Single Regeneration Budget (SRB) and the New Deal for Communities (NDC) [16]. The SRB was launched by the English government in 1994 with the aim to improve the economic, physical and social conditions in local areas [17]. In six years time, over 1000 schemes were funded and 80 % of the total expenditure was spent on the 99 most deprived districts of England [17]. Changes in outcome variables were traced using cross-sectional household surveys and were compared to national trends. At five year follow-up, target areas of the SRB saw larger reductions in the number of residents feeling very unsafe than the rest of England [18]. There were no effects on crime, vandalism, problems with dogs, and litter. However, none of the differences were tested for statistical significance.

The NDC was launched by the English government in 1998 with the aim to reduce the gap between the 39 most deprived urban areas in England and the rest of the country with respect to health, economic, physical and social conditions [19, 20]. Compared to the SRB, the NDC had a more area-based focus by targeting only the most deprived areas of the country. Changes in outcome variables were traced using cross-sectional household surveys and were compared to adjacent, similarly deprived areas. At six year follow-up, target areas of the New Deal for Communities (NDC) saw a 9 % larger improvement in lawlessness and dereliction than similarly deprived areas [19]. This difference was stated to be statistically significant [19]. Target areas also saw significantly larger reductions in criminal victimization, though the relative improvement was only 4 %. There were no effects on fear of crime or feeling unsafe after dark in general. However, positive effects on fear of crime were found in areas with larger safety interventions [20].

The impact of ABIs on perceived area safety may differ between population groups. Safety concerns are more prevalent among women, elderly, ethnic minorities, and individuals with lower socio-economic status [21]. These groups are suggested to be feel more unsafe because of higher physical and social vulnerability, which causes them to feel more at risk of crime [21]. ABIs may be particularly beneficial for these groups, as they try to reduce perceived risk of crime in various ways. To our knowledge, the differential impact of ABIs on perceptions of area safety has been explored only once so far. Contrary to what might be expected, the impact of the NDC on fear of crime at two year follow-up did not significantly differ by gender, age, educational level, and ethnicity [22].

So far, studies on the impact of ABIs on safety perceptions and underlying problems have been limited to England and have paid minimal attention to subgroup differences. Moreover, they have included only a baseline and one follow-up measurement, ignoring trends in outcome over time. An opportunity to address these issues has arisen with the implementation of a Dutch ABI called the “District Approach”. The District Approach was launched by the Dutch government in 2007 with the aim to improve the living conditions in the 40 most deprived districts of the Netherlands. Districts were selected based on objective and subjective measures of physical and socioeconomic deprivation. Interventions were aimed at six main themes: safety, employment, education, housing, the physical environment, and social cohesion. Each district developed a set of locally tailored interventions, which were implemented from mid-2008 onwards.

In the current study we assessed the impact of the District Approach on trends in perceived area safety and underlying problems (perceived physical disorder, perceived social disorder, self-reported criminal victimization) in deprived target districts. A quasi-experimental interrupted time-series design was used. We aimed to assess to what extent trends in perceived area safety and underlying problems changed mid 2008 in the target districts. These trend changes were compared with those in three control groups: rest of the Netherlands, other deprived districts, other deprived districts in the same cities as the target districts. Moreover, we aimed to assess whether results differed by subgroup. We expected to find a more positive trend change in perceived area safety and underlying problems in target districts than in control groups, especially among women, elderly, lower educated people, and target districts with more intensive safety interventions.

## Methods

This study was based on secondary analyses of anonymized survey data. The Medical Ethics Committee of the Academic Medical Centre in Amsterdam, the Netherlands, has confirmed that ethics approval is not necessary, because the Medical Research Involving Human Subjects Act (WMO) does not apply to our study.

### Implementation of the District Approach

**Fig. 1:**
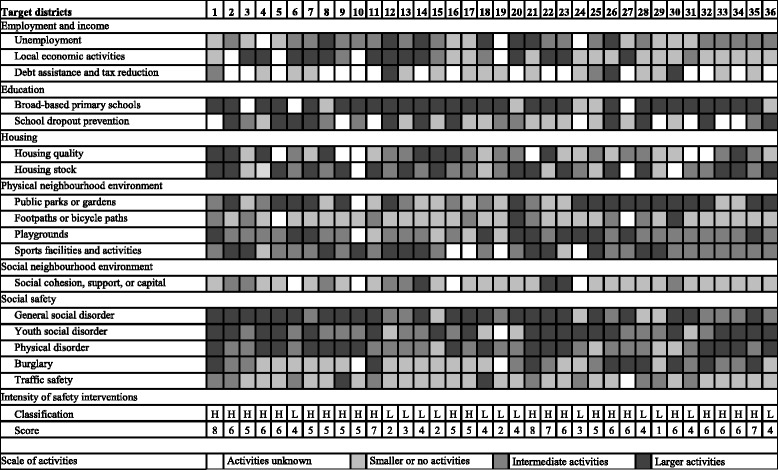
Type and scale of activities carried out in 36 target districts as part of the District Approach (adapted from Droomers et al., 2014 [4])

For 36 out of the 40 target districts, data on the content, duration, and scale of interventions implemented as part of the District Approach since 2008 were retrospectively collected using standardized questionnaires and face-to-face interviews with local district managers [4]. Most target districts addressed all six main themes that were central to the approach. The type and scale of interventions that were implemented to address each main theme varied greatly across the target districts (Fig. 1). Two types of interventions were identified that could potentially improve perceptions of area safety and remove underlying problems. A first group of potentially effective interventions aimed to tackle underlying safety problems like general social disorder, youth social disorder, physical disorder, and burglary. Examples of interventions include extra police surveillance, youth leisure activities, youth counselling, bins, and cleaning services. A second group of potentially effective interventions aimed to improve neighbourhood conditions such as housing quality, housing stock, green space, playgrounds, sports facilities/activities, trails, and social capital. Examples of interventions include demolition of rundown homes, housing renewal, (re)construction of green space and playgrounds, extra sports facilities and activities.

### Data and study population

Repeated cross-sectional data were derived from the National Safety Monitor (NSM) years 2005–2008 and its successor the Integrated Safety Monitor (ISM) years 2008–2011. Both surveys were targeted at non-institutionalized persons of 15 years and older nationwide. The sampling frame was derived from the national population registry. The sampling frame was renewed each year to assure independence of observations, and it was stratified by police region to assure coverage of each Dutch police region. Monthly samples were drawn from the sampling frame using a two-step design, with sub-municipalities in step one and individuals in step two. For NSM, individuals were approached by telephone or interviewer between January and March. For ISM, individuals were sent a letter between mid-September and December in which they were asked to participate by internet or paper-and-pencil survey. Non-respondents were approached by telephone or interviewer. A total of 226,165 individuals were approached between 2005 and 2011. Overall response rate was 62 %. Respondents were excluded when they had no personal identification number (*N* = 269), no zip code information (*N* = 362), or were under 18 years old (*N* = 6609). A total remained of 133,522 adult respondents. Of these respondents, 3,595 resided in the target districts and 12,9927 resided elsewhere in the Netherlands.

### Measures

#### Perceived area safety and underlying problems

Four outcome variables were included:

- *Perceived general safety:* in NSM as well as ISM, respondents were asked whether they sometimes felt unsafe in their own neighbourhood. They could answer yes or no.
- *Perceived physical and social order:* in NSM as well as ISM, respondents were asked whether they judged nine problems to occur often (1), sometimes (2), or (almost) never (3) in their neighbourhood. A physical order variable was computed by averaging the scores on graffiti, litter, dog waste, and demolition of phone booths/bus-cubicles/tram-cubicles. Cronbach’s alpha of the four items was 0.61, indicating fair reliability. A social order variable was computed by averaging the scores on nuisance from youth, nuisance from drugs, nuisance from neighbours, drunken people on the street, and people who get harassed on the street. Cronbach’s alpha of the five items was 0.68, indicating fair reliability. As the distribution of mean scores on both disorder variables was highly skewed, mean scores were dichotomized into ‘disorder generally occurs sometimes or often’ (mean score ≤2) and ‘disorder generally occurs (almost) never’ (mean score >2).
- *Self-reported victimization*: in both NSM and ISM, respondents were asked whether they had been a victim of any of the following fourteen crimes in the past five years: attempted burglary, burglary, bicycle theft, car theft, theft from their car, car damaging, pick pocketing, violent robbery, other thefts, other damaging, sexual abuse, threat of physical abuse, physical abuse, and other crimes. Respondents could answer yes or no. If they answered yes to any of the crimes, they were asked if they were victimized before or after January 1^st^ of last year (NSM), or this year, last year, or earlier (ISM). If respondents were victimized after January 1^st^ of last year (NSM) or this year (ISM) they were asked whether they were last victimized in the own neighbourhood, somewhere else in the municipality, somewhere else in the Netherlands, or in a foreign country. This information was used to compose a dichotomous variable that measured whether or not the respondent had been a victim of one or more crimes after January 1^st^ of last year (NSM) or this year (ISM) in their own neighbourhood.

#### Time variables

The main predictor variable was survey year. We also included the variable survey period, which was dichotomized into ‘pre-intervention period’ (years 2005 to 2008 from the NSM) and ‘intervention period’ (years 2008 to 2011 from the ISM).

#### Districts

The respondents’ district of residence was measured using data on the 4-digit zip codes obtained from the national population registry. The intervention group consisted of the 3,595 respondents living in the 40 target districts. This group comprised 83 zip codes distributed across 18 cities throughout the Netherlands. Nearly three quarter of the zip codes were located in the four largest cities of the Netherlands. Three control groups were included:

1. *Rest of the Netherlands*: consisting of 129,927 respondents living anywhere in the Netherlands but the target districts. This group comprised 3,697 zip codes.
2. *Other deprived districts*: consisting of 11,248 respondents living in districts number 41 to 140 on the official list of most deprived districts of the Netherlands (the target districts are number 1 to 40 on the list). These districts were slightly less deprived than the target districts. This group comprised 257 zip codes distributed across 114 cities and villages throughout the Netherlands. Nearly one quarter of the zip codes were located in the four largest cities of the Netherlands.
3. *Other deprived districts same city*: consisting of 6,022 respondents living in those districts listed under number 2 that were located in the same cities as the target districts. This group comprised 119 zip codes distributed across 18 cities throughout the Netherlands. Over half of the zip codes were located in the four largest cities in the Netherlands.

Because of lower statistical power and the possibility of spillover effects when using the latter two control groups, we used the first group as the main control group.

#### Intensity of safety interventions

In stratified analyses, the intervention group was split based on the intensity of their safety interventions. For 36 out of the 40 target districts, information on intervention content and scale was available to determine programme intensity [4]. First, for each district a list was composed of all interventions that primarily aimed to improve neighbourhood safety by addressing one of four safety related problems: general social disorder, youth social disorder, physical disorder, burglary [4]. Minimum duration was set at one year. Second, for each safety problem, the scale of combined interventions was graded as small (no change expected), intermediate (small changes expected), or large (substantial changes expected). Third, per district, an overall intensity score was calculated by summing the grades for all four safety problems (small = 0, intermediate = 1, large = 2). Target districts with less intensive safety interventions (score <5, *n* = 13) were distinguished from those with more intensive safety interventions (score ≥ 5, *n* = 23). Figure 1 provides an overview of intensity score and intensity classification of the 36 target districts.

#### Covariates

Control variables included age (seven categories: 18–24, 25–34, 35–44, 45–54, 55–64, 65–74, 75 years and older), gender (men, women), ethnicity (ethnic Dutch, non-ethnic Dutch) and educational level (primary-, lower secondary-, higher secondary-, and tertiary level, based on the International Standard Classification of Education).

### Statistical analyses

Interrupted time series analyses were used to assess whether trends in perceived area safety and underlying problems have changed with the implementation of the District Approach in 2008. Multilevel logistic regression models were applied to assess the association between year and any of the outcome variables, i.e., the annual rate of change in the outcome variable. Hereafter, this is called the *trend*. The variable district was included to measure differences in outcome between the target districts and various control groups at the start of the District Approach. The variable period was included to account for any difference in outcome related to the change in survey design in 2008. An interaction term for the variables year and district was included to assess differences in trend between the target districts and various control groups. An interaction term for year and period was included to assess differences in trend between the pre-intervention period and the intervention period. Hereafter, this is called the *trend change*. An interaction term for the variables year, district, and period was included to assess whether trend change varied between the target districts and various control groups.

All analyses were controlled for age, gender, ethnicity and education. Additional analyses were stratified by gender (men versus women), age (under 55 years old versus 55 years and older), education (primary- and lower secondary level versus higher secondary- and tertiary level), and intensity of the safety interventions (less intensive interventions versus more intensive interventions). Multilevel regression analyses were applied to take into account clustering of respondents in districts. Level 1 represented individuals and level 2 represented zip codes. All analyses were carried out using STATA 11.0 software. Statistical significance was set at 0.05.

## Results

**Fig. 2:**
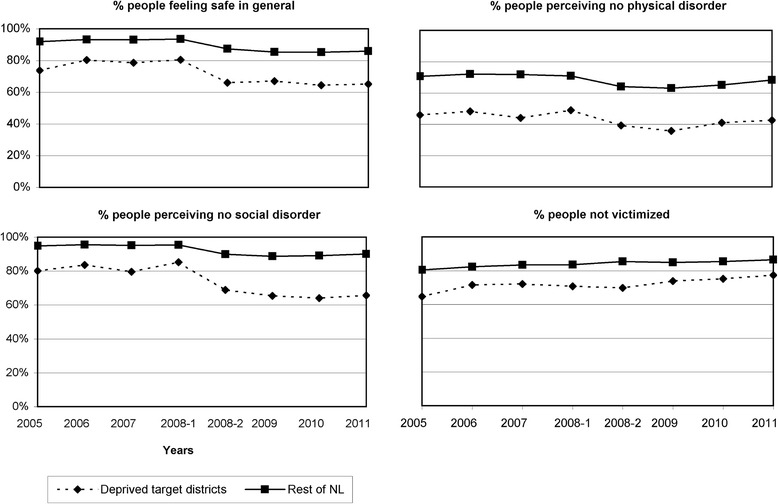
Trend in perceived area safety and underlying problems in target districts and the rest of the Netherlands

Adults in target districts were more often under 35 years old, of non-Dutch origin, and lower educated compared to adults in all control groups (Table 1). Moreover, they reported lower levels of general safety, physical order, social order, and non-victimization compared to adults in the rest of the Netherlands (Fig. 2). Prevalence of all four outcome variables remained relatively stable over time in both groups, with two exceptions. First, between the first and second half of 2008, both groups showed a sharp decline in the number of people feeling generally safe and perceiving order. Second, after the implementation of the District Approach, target districts showed a small increase in non-victimization.

**Table 1:** Characteristics of the study population

	Target districts	Control groups		
		Rest of the Netherlands	Deprived districts	Deprived districts, same city
Numbers				
* n* 4-digit zipcodes	83	3,697	257	119
* n* individuals in total	3,595	129,927	11,248	6,022
Characteristics^a^				
Age (%)				
15 − 24 years old	14.6	9.2	12.7	14.2
25 − 34 years old	21.2	13.5	18.2	20.4
35 − 44 years old	17.6	19.8	17.8	18.3
45 − 54 years old	16.9	20.0	16.6	15.4
55 − 64 years old	13.4	18.5	15.8	14.6
65 − 74 years old	9.5	11.9	11.1	9.6
75 years and older	6.8	7.1	7.8	7.5
Gender (%)				
Women	52.6	52.0	52.4	52.5
Men	47.4	48.0	47.6	47.5
Ethnicity (%)				
Ethnic Dutch	60.6	88.4	80.0	76.6
Non-ethnic Dutch	39.2	11.5	20.0	23.3
Education (%)				
Primary level	36.0	26.0	28.4	24.6
Lower secondary level	8.2	9.9	8.9	7.8
Higher secondary level	26.8	32.5	29.7	27.8
Tertiary level	21.8	25.3	26.7	33.3

^a^Characteristics represent mean values for years 2005 to 2011

Tables 2, 3 and 4 show the results of the regression analyses. Table 2 displays the trends in target districts versus the rest of the Netherlands. For general safety, the trend in target districts changed from a nearly significantly positive trend in the pre-intervention period into a flat trend in the intervention period. This negative trend change was not statistically significant. A similar but significantly negative trend change was found in the rest of the Netherlands. As a result, there was no difference in trend change between target districts and the rest of the Netherlands. For physical order, target districts and the rest of the Netherlands showed similar slightly positive trend changes, though the trend change was only statistically significant in the latter group. For social order, target districts showed a slightly negative trend change, while the rest of the Netherlands showed a slightly positive trend change. Thus, target districts showed a more negative trend change than in the rest of the Netherlands. However, neither the trend changes themselves, nor the between-district differences in trend change were statistically significant. For non-victimization, target districts showed a positive trend change, while the rest of the Netherlands showed a slightly negative trend change. Thus, target districts showed a more positive trend change than the rest of the Netherlands. Even though the trend changes themselves were not statistically significant, between-district differences in trend change were nearly significant. Overall, adjustment for individual-level demographic and socio-economic factors did not alter the results.

**Table 2:** Trends in perceived area safety and related problems in target districts and the rest of the Netherlands (NL)

		Trend (regression coefficient ß (95 % Confidence Interval))		
		Pre intervention	Intervention	Intervention versus pre intervention period
		(2005–2008)	(2008–2011)	
General safety				
M1^a^	Target districts (A)	0.08 (−0.00 – 0.15)	−0.01 (−0.10 – 0.07)	−0.09 (−0.23 – 0.05)
	Rest of NL (B)	0.05 (0.02 – 0.09)*	−0.04 (−0.06 – -0.02)*	−0.09 (−0.13 – -0.05)*
	A versus B			0.00 (−0.14 – 0.15)
M2^b^	Target districts (A)	0.08 (−0.00 – 0.16)	−0.00 (−0.09 – 0.08)	−0.08 (−0.22 – 0.06)
	Rest of NL (B)	0.05 (0.02 – 0.08)*	−0.03 (−0.05 – -0.01)*	−0.08 (−0.12 – -0.04)*
	A versus B			0.00 (−0.15 – 0.15)
Physical order				
M1^a^	Target districts (A)	0.00 (−0.06 – 0.07)	0.06 (−0.01 – 0.14)	0.06 (−0.07 – 0.19)
	Rest of NL (B)	−0.01 (−0.03 – 0.00)	0.07 (0.06 – 0.09)*	0.09 (0.06 – 0.11)*
	A versus B			−0.03 (−0.16 – 0.10)
M2^b^	Target districts (A)	0.00 (−0.07 – 0.07)	0.07 (−0.01 – 0.15)	0.07 (−0.06 – 0.20)
	Rest of NL (B)	−0.01 (−0.03 – 0.00)	0.08 (0.07 – 0.10)*	0.09 (0.07 – 0.12)*
	A versus B			−0.02 (−0.16 – 0.11)
Social order				
M1^a^	Target districts (A)	0.03 (−0.05 – 0.11)	−0.03 (−0.12 – 0.05)	−0.06 (−0.21 – 0.08)
	Rest of NL (B)	0.00 (−0.04 – 0.04)	0.01 (−0.01 – 0.03)	0.01 (−0.04 – 0.06)
	A versus B			−0.07 (−0.22 – 0.08)
M2^b^	Target districts (A)	0.03 (−0.06 – 0.11)	−0.02 (−0.10 – 0.07)	−0.04 (−0.19 – 0.10)
	Rest of NL (B)	−0.00 (−0.04 – 0.04)	0.02 (−0.00 – 0.05)	0.03 (−0.02 – 0.07)
	A versus B			−0.07 (−0.22 – 0.08)
Non-victimization				
M1^a^	Target districts (A)	0.01 (−0.06 – 0.08)	0.10 (0.02 – 0.19)*	0.09 (−0.05 – 0.23)
	Rest of NL (B)	0.06 (0.04 – 0.08)*	0.04 (0.02 – 0.06)*	−0.02 (−0.05 – 0.01)
	A versus B			0.11 (−0.03 – 0.26)
M2^b^	Target districts (A)	0.01 (−0.06 – 0.08)	0.11 (0.02 – 0.20)*	0.10 (−0.04 – 0.24)
	Rest of NL (B)	0.05 (0.03 – 0.07)*	0.03 (0.01 – 0.05)*	−0.02 (−0.05 – 0.01)
	A versus B			0.12 (−0.02 – 0.27)

*P ≤ 0.05

^a^Unadjusted model

^b^Adjusted for age, gender, ethnicity, and education

**Table 3:** Trends in perceived area safety and underlying problems in target districts and various control groups

	Trend^a^ (regression coefficient ß (95 % Confidence Interval))		
	Pre intervention	Intervention	Intervention versus pre intervention
	(2005–2008)	(2008–2011)	
General safety			
Target districts (A)	0.08 (−0.00 – 0.16)	−0.00 (−0.09 – 0.08)	−0.08 (−0.22 – 0.06)
Rest of NL (B)	0.05 (0.02 – 0.08)*	−0.03 (−0.05 – -0.01)*	−0.08 (−0.12 – -0.04)*
A versus B			0.00 (−0.15 – 0.15)
Deprived districts (C)	−0.00 (−0.08 – 0.08)	−0.02 (−0.08 – 0.04)	−0.02 (−0.12 – 0.09)
A versus C			−0.04 (−0.21 – 0.13)
Deprived districts, same city (D)	0.03 (−0.07 – 0.12)	0.01 (−0.07 – 0.08)	−0.02 (−0.15 – 0.11)
A versus D			−0.04 (−0.22 – 0.15)
Physical order			
Target districts (A)	0.00 (−0.07 – 0.07)	0.07 (−0.01 – 0.15)	0.07 (−0.06 – 0.20)
Rest of NL (B)	−0.01 (−0.03 – 0.00)	0.08 (0.07 – 0.10)*	0.09 (0.07 – 0.12)*
A versus B			−0.02 (−0.16 – 0.11)
Deprived districts (C)	−0.01 (−0.07 – 0.04)	0.06 (−0.01 – 0.10)	0.07 (−0.00 – 0.15)
A versus C			0.01 (−0.14 – 0.16)
Deprived districts, same city (D)	−0.01 (−0.09 – 0.06)	0.06 (0.00 – 0.13)*	0.08 (−0.02 – 0.18)
A versus D			0.01 (−0.15 – 0.17)
Social order			
Target districts (A)	0.03 (−0.06 – 0.11)	−0.02 (−0.10 – 0.07)	−0.04 (−0.19 – 0.10)
Rest of NL (B)	−0.00 (−0.04 – 0.04)	0.02 (−0.00 – 0.05)	0.03 (−0.02 – 0.07)
A versus B			−0.07 (−0.22 – 0.08)
Deprived districts (C)	−0.01 (−0.10 – 0.07)	−0.06 (−0.12 – -0.00)*	−0.05 (−0.16 – 0.06)
A versus C			0.02 (−0.15 – 0.20)
Deprived districts, same city (D)	0.04 (−0.06 – 0.14)	−0.06 (−0.14 – 0.01)	−0.10 (−0.23 – 0.03)
A versus D			0.07 (−0.12 – 0.26)
Non-victimization			
Target districts (A)	0.01 (−0.06 – 0.08)	0.11 (0.02 – 0.20)*	0.10 (−0.04 – 0.24)
Rest of NL (B)	0.05 (0.03 – 0.07)*	0.03 (0.01 – 0.05)*	−0.02 (−0.05 – 0.01)
A versus B			0.12 (−0.02 – 0.27)
Deprived districts (C)	0.01 (−0.05 – 0.08)	0.02 (−0.04 – 0.07)	0.01 (−0.09 – 0.09)
A versus C			0.09 (−0.07 – 0.25)
Deprived districts, same city (D)	0.07 (−0.01 – 0.15)	−0.01 (−0.08 – 0.06)	−0.08 (−0.20 – 0.03)
A versus D			0.17 (−0.01 – 0.35)

*P ≤ 0.05

^a^Trend represents the yearly change in ln(odds) of safety, adjusted for age, gender, ethnicity, and education

**Table 4:** Trends in self-reported non-victimization in target districts and the rest of the Netherlands; stratified by subgroup

		Trend in non-victimization^a^ (regression coefficient ß (95 % Confidence Interval))		
		Pre intervention	Intervention	Intervention versus pre intervention
		(2005–2008)	(2008–2011)	
Gender				
Men	Target districts (A)	0.07 (−0.03 – 0.18)	0.04 (−0.09 – 0.17)	−0.03 (−0.24 – 0.17)
	Rest of NL (B)	0.04 (0.01 – 0.07)*	0.04 (0.01 – 0.07)*	0.00 (−0.04 – 0.04)
	A versus B			−0.03 (−0.23 – 0.18)
Women	Target districts (A)	−0.04 (−0.14 – 0.06)	0.18 (0.05 – 0.30)*	0.22 (0.02 – 0.41)*
	Rest of NL (B)	0.06 (0.03 – 0.10)*	0.02 (−0.01 – 0.05)	−0.04 (−0.09 - -0.00)*
	A versus B			0.26 (0.06 – 0.46)*
Age				
Younger	Target districts (A)	0.04 (−0.04 – 0.12)	0.04 (−0.06 – 0.14)	−0.00 (−0.16 – 0.16)
	Rest of NL (B)	0.06 (0.03 – 0.08)*	0.03 (0.00 – 0.05)*	−0.03 (−0.06 – 0.01)
	A versus B			0.03 (−0.13 – 0.19)
Older	Target districts (A)	−0.09 (−0.25 – 0.06)	0.38 (0.18 – 0.58)*	0.47 (0.17 – 0.78)*
	Rest of NL (B)	0.06 (0.01 – 0.10)*	0.05 (0.01 – 0.08)*	−0.01 (−0.07 – 0.05)
	A versus B			0.48 (0.17 – 0.80)*
Educational level				
Lower	Target districts (A)	−0.10 (−0.21 – 0.01)	0.21 (0.07 – 0.36)*	0.31 (0.09 – 0.54)*
	Rest of NL (B)	0.04 (−0.00 – 0.08)	0.01 (−0.02 – 0.05)	−0.02 (−0.08 – 0.03)
	A versus B			0.34 (0.10 – 0.57)*
Higher	Target districts (A)	0.12 (0.02 – 0.22)*	0.06 (−0.06 – 0.17)	−0.06 (−0.25 – 0.13)
	Rest of NL (B)	0.06 (0.03 – 0.09)*	0.04 (0.01 – 0.06)*	−0.02 (−0.06 – 0.02)
	A versus B			−0.04 (−0.23 – 0.15)
Intensity of safety interventions				
Lower	Target districts (A)	−0.07 (−0.19 – 0.06)	0.12 (0.03 – 0.27)	0.18 (−0.06 – 0.43)
	Rest of NL (B)	0.05 (0.03 – 0.07)*	0.03 (0.01 – 0.05)*	−0.02 (−0.06 – 0.01)
	A versus B			0.21 (−0.04 – 0.46)
Higher	Target districts (A)	0.08 (−0.02 – 0.17)	0.09 (−0.03 – 0.20)	0.01 (−0.18 – 0.20)
	Rest of NL (B)	0.05 (0.03 – 0.07)*	0.03 (0.01 – 0.05)*	−0.02 (−0.06 – 0.01)
	A versus B			0.03 (−0.16 – 0.22)

*P ≤ 0.05

^a^Trend represents the yearly change in ln(odds) of non-victimization, adjusted for age, and/or ethnicity, and/or education

Table 3 compares the trends in target districts with those in three different control groups. Overall, results were similar across control groups. For general safety, the slightly negative trend change in target districts was similar to that in the rest of the Netherlands, but somewhat more negative than in both groups of other deprived districts. For physical order, the slightly positive trend change in target districts was similar to that in all control groups. For social order, the somewhat negative trend change in target districts was slightly more negative than in the rest of the Netherlands, but somewhat more positive than in both groups of other deprived districts, especially those located in the same city as target districts. For non-victimization, the somewhat positive trend change in target districts was somewhat more positive than in all control groups. None of the between-district differences in trend change were statistically significant. However, for non-victimization, differences with the rest of the Netherlands and other deprived districts in the same city were nearly significant.

**Fig. 3:**
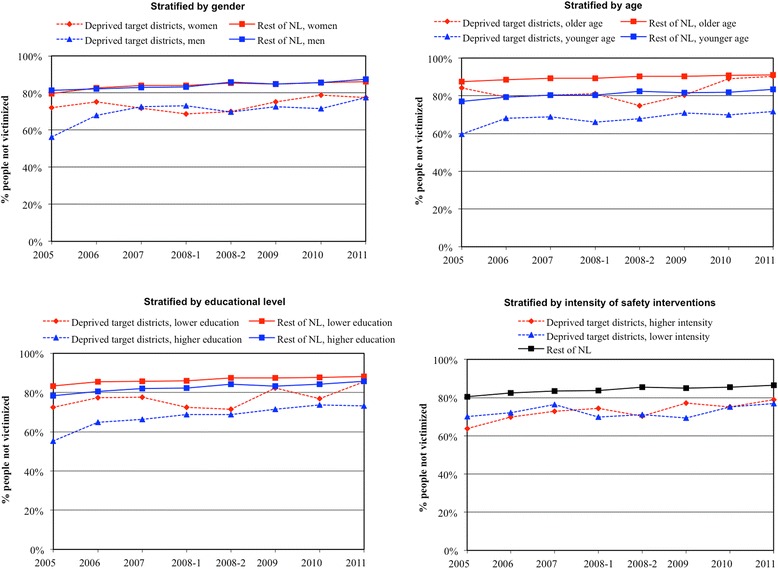
Trend in non-victimization in target districts and the rest of the Netherlands, stratified by gender, age, educational level and intensity of safety interventions

Table 4 displays the non-victimization trends in target districts versus the rest of the Netherlands, stratified by subgroup. Women, older adults, and lower educated adults living in target districts showed a statistically significantly more positive trend change than those living in the rest of the Netherlands. Figure 3 shows that for these subgroups, area-based inequalities in victimization widened before the implementation of the District Approach and narrowed afterwards. Target districts with less intensive safety interventions showed a somewhat more positive trend change than the rest of the Netherlands, but this difference was not significant. There were no between-district differences in trend change for men, younger adults, higher educated adults, and target districts with more intensive safety interventions. For the other safety indicators, there were no significant between-district differences in trend change for any of the subgroups, though some groups showed slight indications of differences (results not shown). Patterns were inconsistent across safety indicators. For general safety, between-district trend change differences were somewhat negative for men, but somewhat positive for women. For physical order, between-district trend change differences were slightly negative for women and lower educated adults. For social order, between-district trend change differences were somewhat negative for men, older adults, and lower educated adults.

## Discussion

Compared to the national average, a lower percentage of adults in target districts felt generally safe, perceived physical and social order, and were not victimized. These differences hardly changed with the implementation of the District Approach in the target districts, starting in 2008. However, the proportion of non-victimized people increased somewhat more in target districts than in the rest of the Netherlands or in other deprived districts. These between-district differences were significant for women, older adults, and lower educated adults only.

### Limitations

In a natural experiment like the District Approach, individuals are not randomly allocated to an intervention or control group. As a result, the two groups may differ at baseline in ways related to the study outcome. We tried to reduce this bias in ways recommended by the Medical Research Council guidance for evaluating natural experiments [23]. To account for possible unobservable group differences, we adopted a quasi-experimental time-series design in which changes over time are compared between the intervention and control group. To account for possible observable group differences, we included matched control groups and we adjusted our analyses for various observable demographic and socio-economic characteristics.

An important condition for a quasi-experimental time-series design is that the composition of both groups remains stable over time [23]. In our study, the use of repeated cross-sectional data may have caused variations in group compositions over time in two ways. First, our *sample* may have varied over time. However, given that the same sampling design was used each year, there is little reason to expect the variation to be systematic. Second, the *source population* for the sample may have varied over time as a result of selective migration. Adults that have benefited from the District Approach, for example by acquiring new skills that allow access to better jobs, may have moved out of the target districts. If movers experienced better perceived area safety than the ones staying behind, this may have caused us to underestimate the safety impact of the District Approach. However, there were no indications of such selective migration patterns in the target districts. The number of people moving up the socio-economic ladder and the number of those moving out of their neighbourhood did not increase after implementation of the District Approach [24]. Evaluation studies of the NDC also failed to find indications of selective migration effects. There was no significant association between residential mobility and change in safety concerns [25] and panel data yielded similar changes in safety outcomes as the use of repeated cross-sectional data [19, 26]. Moreover, to take possible variations in group composition into account, we adjusted our analyses for various demographic and socio-economic factors.

Total non-response was nearly 40 %. A comparison of weighted and non-weighted characteristics of the total sample revealed a small overrepresentation of people over 45 years old, women, ethnic Dutch, and higher educated people (data not shown). Even though the population of non-respondents did not appear to be selective in socio-demographic terms, it may have been selective in other ways related to our study outcome. However, this will only bias our results if selectivity changed over time, which we perceive to be unlikely.

In 2008, the main survey mode of the safety monitor changed from telephone to internet. This may explain the sharp decreases in 2008 in the number of people that reported feeling safe and that perceived physical or social order (Fig. 2). Adults who participated by internet reported more disorder than those who participated by telephone [27]. When interviewed by telephone, people may be more inclined to give socially desirable answers or to choose the last mentioned option of “no disorder” [28]. To take the change in survey design into account, we have controlled all our regression analyses for the variable period. For all safety indicators except non-victimization, period appeared to be a confounder.

### Interpretation of results

Results of our study are generally in line with a recent non peer-reviewed evaluation of the District Approach [24]. Using a regression discontinuity design, the researchers concluded that there was no demonstrable overall positive effect of the District Approach on perceived area safety or underlying problems. Using a different and more elaborate design, results of our study were consistent with these results. In the peer-reviewed literature, only two ABIs have been evaluated for their safety impact. Like the Dutch District Approach, the English SRB appeared to have had only limited impact on safety concerns and underlying problems [18]. Evaluations of the English NDC, on the other hand, indicated positive effects on underlying problems of safety concerns, with improvements in victimization and especially in perceived order [19].

The NDC study may have yielded a larger number of positive results than the current study because of an almost twice as long follow-up time. Perhaps, area safety – especially perceptions of area safety – needs more time to change in response to ABIs. However, an NDC evaluation found safety changes to be larger at two year follow-up than at four and six year follow-up, suggesting safety effects to be visible already at the short term [29]. An alternative explanation for the larger number of positive results in the NDC study may be the larger sample size [30]. Therefore, they might have had more power to also detect small safety effects. Finally, differences in results may have been due to differences in the content of the ABIs. The NDC and the District Approach have both invested in the same problems (employment, education, housing, the physical environment, safety, and social cohesion), and both have given each area the autonomy to develop its own set of tailored interventions. However, the NDC did seem to have made larger investments with respect to social cohesion and crime than the District Approach [4, 19].

We did not find a general improvement in perceptions of general safety or disorder in the target districts. One reason for this lack of change may be that the target districts varied greatly with respect to the interventions that were implemented and the context in which they were implemented [Fig. 1]. Specific interventions may have successfully improved perceptions of safety or disorder in specific contexts, but analyses of all 40 target districts combined may have concealed these successes. Unfortunately, due to lack of statistical power, we were unable to analyse changes in each target district separately. Another possible reason for a lack of improvement in perceptions of general safety in specific, is that the interventions might have had opposite effects on people’s perceptions. For example, security measures such as locks and fences may cause people to feel more protected [14], but it may also create an unpleasant and hostile environment and make people more aware of the threats in the area, causing them to feel unsafe [8]. In a similar way, stronger social networks may cause people to feel less vulnerable to crime, but may also increase communication about events of crime [7, 8, 13].

There were indications of improvements in more objective safety problems, i.e., the prevalence of non-victimization. However, improvements were only visible among women, older adults, and lower educated adults, and not among their counterparts. On the one hand, we should recognise the possibility that observed subgroup differences may be attributable to chance, resulting from the large number of subgroup differences tested (type I error). On the other hand, these subgroup differences may be the result of differences in neighbourhood exposure. In Dutch society, women spend more time taking care of the children and doing household chores than men [31]. Dutch older adults and lower educated adults are less often employed than their counterparts [31]. As a result, these population groups may spend more time in their local neighbourhood.

## Conclusions

The current study provides limited evidence to suggest that ABIs may improve health in deprived areas by improving key social determinants of health such as area safety. At least at the short term, the Dutch District Approach was not followed by improvements in perceived area safety or perceived disorder in the deprived target districts. However, though we recognise that the strength of evidence may be limited, there were indications of a somewhat positive impact of the District Approach on more objective safety outcomes, that is, the number of crime victims in deprived target areas. More evaluation studies are needed to fully determine ABIs’ potential to address safety issues in deprived areas. Quasi-experimental research as presented in this article should be complemented with research that aims to identify the mechanisms and conditions for change. Such studies may provide insight into why and under which conditions ABIs are able to improve area safety in deprived areas.
